# A description of the methods of the aspirin supplementation for pregnancy indicated risk reduction in nulliparas (ASPIRIN) study

**DOI:** 10.1186/s12884-017-1312-x

**Published:** 2017-05-03

**Authors:** Matthew K. Hoffman, Shivaprasad S. Goudar, Bhalachandra S. Kodkany, Norman Goco, Marion Koso-Thomas, Menachem Miodovnik, Elizabeth M. McClure, Dennis D. Wallace, Jennifer J. Hemingway-Foday, Antoinette Tshefu, Adrien Lokangaka, Carl L. Bose, Elwyn Chomba, Musaku Mwenechanya, Waldemar A. Carlo, Ana Garces, Nancy F. Krebs, K. Michael Hambidge, Sarah Saleem, Robert L. Goldenberg, Archana Patel, Patricia L. Hibberd, Fabian Esamai, Edward A. Liechty, Robert Silver, Richard J. Derman

**Affiliations:** 1 Christiana Care, Newark, DE USA; 2 KLE’s JN Medical College, Belgaum, India; 3 RTI International, 3040 E. Cornwallis Road, Research Triangle Park, NC USA; 4 Eunice Kennedy Shriver National Institute of Child Health and Human Development, Bethesda, MD USA; 5 Kinshasa School of Public Health, Kinshasa, Democratic Republic of the Congo; 6 University of North Carolina, Chapel Hill, NC USA; 7 University Teaching Hospital, Lusaka, Zambia; 8 University of Alabama at Birmingham, Birmingham, AL USA; 9 Instituto de Nutrición de Centroamérica y Panamá (INCAP), Guatemala City, Guatemala; 10 University of Colorado School of Medicine, Denver, CO USA; 11 Aga Khan University, Karachi, Pakistan; 12 Columbia University, New York, NY USA; 13 Lata Medical Research Foundation, Nagpur, India; 14 Boston University School of Public Health, Boston, MA USA; 15 Department of Child Health and Paediatrics, Moi University School of Medicine, Eldoret, Kenya; 16 School of Medicine, Indiana University, Indianapolis, IN USA; 17 University of Utah, Salt Lake City, UT USA; 18 Thomas Jefferson University, Philadelphia, PA USA

**Keywords:** Prematurity, Preterm birth, Low dose Aspirin

## Abstract

**Background:**

Preterm birth (PTB) remains the leading cause of neonatal mortality and long term disability throughout the world. Though complex in its origins, a growing body of evidence suggests that first trimester administration of low dose aspirin (LDA) may substantially reduce the rate of PTB.

**Methods:**

Hypothesis: LDA initiated in the first trimester reduces the risk of preterm birth.

Study Design Type: Prospective randomized, placebo-controlled, double-blinded multi-national clinical trial conducted in seven low and middle income countries. Trial will be individually randomized with one-to-one ratio (intervention/control)

Population: Nulliparous women between the ages of 14 and 40, with a singleton pregnancy between 6 0/7 weeks and 13 6/7 weeks gestational age (GA) confirmed by ultrasound prior to enrollment, no more than two previous first trimester pregnancy losses, and no contraindications to aspirin.

Intervention: Daily administration of low dose (81 mg) aspirin, initiated between 6 0/7 weeks and 13 6/7 weeks GA and continued to 36 0/7 weeks GA, compared to an identical appearing placebo. Compliance and outcomes will be assessed biweekly.

**Outcomes:**

*Primary outcome*: Incidence of PTB (birth prior to 37 0/7 weeks GA).

*Secondary outcomes* Incidence of preeclampsia/eclampsia, small for gestational age and perinatal mortality.

**Discussion:**

This study is unique as it will examine the impact of LDA early in pregnancy in low-middle income countries with preterm birth as a primary outcome. The importance of developing low-cost, high impact interventions in low-middle income countries is magnified as they are often unable to bear the financial costs of treating illness.

**Trial registration:**

ClinicalTrials.gov identifier: NCT02409680 Date: March 30, 2015

## Background

Preterm delivery, defined as delivery prior to 37 weeks 0/7 days gestation, remains the dominant cause of neonatal morbidity and mortality throughout the world [1–3] and directly leads to 28% of neonatal deaths within the first seven days of life [4]. Moreover it is responsible for up to 50% of pediatric neurodevelopmental disorders [5]. Infants born prematurely are also at increased risk for long term medical complications such as respiratory, gastrointestinal, cardiovascular, metabolic, and neurodevelopmental disorders [6, 7].

The risk of preterm birth is highest in low-middle income countries where an estimated 12% of births are preterm compared to 5-7% in high-income countries [8]. Of the near 13 million preterm births worldwide in 2005, 11 million were in Africa and Asia [9]. Given the tremendous medical, financial and emotional burden of preterm birth in the developing world and the limited resources to provide neonatal care, any interventions with the potential to reduce the rate of preterm birth deserve consideration. An ideal intervention to reduce preterm birth would be one that is widely available, inexpensive, and safe for the mother and fetus. Low dose aspirin (LDA) may be just such an intervention.[6, 7]

### Biology of aspirin in pregnancy

Aspirin is best known for its analgesic properties; however, it is well documented that aspirin is an anti-inflammatory and potent inhibitor of platelet aggregation, contributing to its anti-thrombotic effects. Both inflammation and thrombosis are implicated in pathways responsible for many cases of preterm birth, preeclampsia and fetal growth restriction. The primary biologic effects of aspirin are mediated by inhibition of the enzyme cyclooxygenase (Cox) [10–12], which produces substances known to be involved in the defined pathways of spontaneous preterm birth and placental dysfunction (preeclampsia/growth restriction/stillbirth) [13]. By decreasing these mediators and in turn decreasing inflammation and placental dysfunction due to thrombosis, aspirin may reduce the rates of the major obstetrical complications of preterm birth, preeclampsia and growth restriction.

The time frame in which LDA is initiated appears to be important. Biologically it has been demonstrated that the process of preterm birth in many cases begins prior to 16 weeks [13]. Likewise, placental invasion of the maternal decidua occurs in the first trimester and underlies the pathologic processes of preeclampsia and pathologic growth restriction. Thus, aspirin may be maximally effective if initiated in the first trimester. Indeed, this is supported by available data (see below).

### Aspirin and preterm birth

Though available evidence is promising, data regarding the early use of low-dose aspirin (LDA) in pregnancy to prevent preterm birth are limited. A single trial of preconception aspirin and meta- analyses that examine the subset of women in larger studies that have begun LDA 16 weeks or earlier, have shown a persistent impact on preterm birth. For example, Roberge et al., in a meta-analysis of LDA included 22 trials that included 11,302 women, found a 19% reduction in the overall rate of preterm birth (RR 0.81, 95% CI 0.71 to 0.92) [14]. However when the analysis was restricted to women who initiated aspirin prior to 16 0/7 weeks, a 65% reduction was observed (6 trials including 904 women, RR 0.35, 95% CI to 0.22 to 0.57).

A trial of preconception LDA, the Effects of Aspirin in Gestation and Reproduction (EAGeR) trial shows a similar trend towards risk reduction in a cohort of 1,078 women who were randomized to LDA prior to conception. If only the original cohort (women with just one prior loss or less) is examined, there is a 55% reduction in preterm birth (RR 0.45; p-value of 0.087; CI 0.19 to 1.08). When expanded to include the entire cohort (includes women with more than one fetal loss), there is a 28% risk reduction (RR of 0.72; p value 0.260). The lack of significant difference in this trial is not surprising given the small number of women with PTB but the trend is encouraging [15], a reduction in the rate of PTB between 28% and 65%.

### Aspirin and preeclampsia

Many large randomized controlled clinical trials have examined the efficacy of LDA in preventing preeclampsia. Duley and colleagues performed a systematic review that included 51 trials involving 36,500 women treated with antiplatelet agents for the prevention of preeclampsia [16]. Forty-four of these trials involved the use of aspirin alone (compared with placebo or no treatment) while the remainder included other treatments, often in conjunction with aspirin. Overall, the use of antiplatelet agents conferred a 19% reduction in the risk of preeclampsia (RR 0.81; 95% CI, 0.74-0.96). There was a greater risk reduction in women treated with doses greater than 75 mg/day (RR 0.49, 95% CI 0.38-0.65) compared to lower doses (RR 0.86, 95% CI 0.79-0.93). Other reviews emphasize the increased benefit from LDA in women with historical risk factors (high risk for preeclampsia) and suggest that focusing on at risk groups would decrease the number of women it is necessary to treat to prevent a single case of preeclampsia [17–19]. As with preterm birth, the effect size increases when LDA is initiated earlier in gestation. A meta-analysis of 33 trials reported a risk reduction of 0.62 (95% CI 0.49 to 0.78); however, when this was restricted to women who began therapy <16 weeks the effect size was greater (RR 0.47, 95% CI 0.36 to 0.62). Of course, the “risk: benefit” ratio also is influenced by risk, which appears to be quite low for LDA. Thus, treatment of low risk women may be justified.

### Aspirin and growth restriction (small for gestational Age)

Low dose aspirin also may reduce the risk of other adverse perinatal outcomes such as fetal growth restriction and late fetal death. In 32 trials of 24,310 women, antiplatelet therapy conferred an 8% reduction in SGA (RR 0.92, 95% CI 0.85-1.00) in women treated with the intent to prevent preeclampsia [20]. LDA also has not been effective when started after the diagnosis of preeclampsia or diagnosis of a growth restricted fetus [21–23]. Once again, early initiation may be the key to success. A meta-analysis reported a 14% reduction in fetal growth restriction among women treated with LDA (RR 0.86, 95% CI 0.75-99); however when only women who initiated therapy at or before 16 weeks were examined the effect size was 54% (RR 0.46, 95% CI 0.33-0.66) [14].

The review by Duley and colleagues noted a 16% reduction in combined fetal, neonatal, and infant mortality in women taking antiplatelet therapy (RR 0.84, 95% CI 0.74-0.96) [24]. Perinatal death and SGA fetuses were not primary end points of these trials.

### Aspirin safety

LDA is attractive as a potential therapy for reproductive disorders because it has a demonstrated track record of both fetal and maternal safety. Randomized controlled trials (RCTs) in thousands of women showed no increase in adverse fetal sequelae in doses < 150 mg per day [22, 24]. In a meta-analyses of 22 studies, aspirin was not associated with an overall increase in the risk of congenital malformations [25]. The same meta-analysis reported an increase in the risk of gastroschisis (OR 2.37; 95% CI, 1.44 – 3.88) in infants exposed to high-dose aspirin (325 mg per day) in the first trimester [25]. This is biologically plausible since this malformation may be caused by vascular disruption of mesenteric vessels [26]. Results were not confirmed in a recent population based case-control study [27, 28] and the cause and effect between low dose aspirin use and gastroschisis remains uncertain. Studies regarding safety are difficult to compare due to different doses, duration, and timing of aspirin use (with regard to pregnancy). Nonetheless, the majority of data indicates minimal fetal risk from *in utero* LDA exposure. In fact, a recent study noted that LDA was actually associated with a reduction in neurobehavioral difficulties in very preterm infants [29].

In terms of maternal safety, a metal-analyses of 22,760 women found no difference in the rate of postpartum hemorrhage amongst women using LDA and placebo [30]. Similarly the rates of abruption and discontinuation were similar between LDA and placebo [30].

### Summary

We intend to study the effects of LDA in nulliparous women for several reasons. First, although it would be of interest to study the effect of LDA in women at high risk for preterm birth (e.g., prior preterm birth), such women may undergo interventions intended to decrease their risk of preterm birth. Thus, a study in patients with prior preterm birth would have numerous potential confounders such as the use of progesterone, cervical cerclage and closer monitoring. Conversely, multiparous women with prior term births would be at very low risk for preterm birth and would also be a suboptimal population to study. Nulliparous women appear to be an ideal population since they will not undergo special interventions in an attempt to avoid preterm birth. Also, it appears that the risk of preterm birth in nulliparous women is higher than for the general obstetric population [31, 32]. In summary, available data suggest that LDA may be a safe, widely available and inexpensive intervention that may significantly reduce the risk of preterm birth. However, this possibility needs to be proven in a properly designed RCT with preterm birth as the primary outcome. Such a clinical trial in a racially, ethnically and geographically diverse population could best be accomplished through the established infrastructure of the Global Network for Women’s and Children’s Health Research (Global Network), a multi-country research network with study sites in India, Pakistan, Guatemala, Kenya, Zambia and the Democratic Republic of Congo [33].

## Methods/design

### Study hypothesis

This trial has the following study hypotheses:

### Primary hypothesis or question

Our primary hypothesis is that nulliparous women with no more than two previous first trimester pregnancy losses who are treated with LDA daily beginning between 6 0/7 weeks and 13 6/7 weeks GA through 36 0/7 weeks GA will reduce the rate of preterm birth from all causes by 20%.

### Secondary hypothesis

Women who take antenatal daily LDA initiated at 6 0/7 to 13 6/7 weeks GA will have lower rates of:

- Small for gestational age infants
- Eclampsia and preeclampsia
- Perinatal Mortality

We propose a randomized, placebo-controlled, double-blinded multicenter clinical trial to assess the efficacy of LDA in the reduction of preterm birth. Women will be randomized equally to receive either daily LDA (81 mg) or an identical appearing placebo beginning between 6 0/7 weeks and 13 6/7 weeks GA and continuing until 36 0/7 weeks GA or delivery.

### Study Population

A total of 11,920 nulliparous women will be enrolled (5960 per group) across seven sites in sub-Saharan Africa, South Asia, and Latin America. For balance, each site will enroll no more than 25% of the total sample. To study the possible effect of LDA on anemia, we will enroll 500 women in a sub-study, with each site contributing at least 10% of the sample.

### Inclusion criteria

Inclusion criteria include the following women who are residents of the study areas:

- Nulliparous women between 18 – 40 years of age (Minors who are ≥ 14 years of age may be enrolled if permitted by the country’s ethical guidelines.);
- No more than two previous first trimester pregnancy losses;
- No medical contraindications to aspirin;
- Single live intrauterine pregnancy (IUP) between 6 0/7 and 13 6/7 weeks GA corroborated by an early dating ultrasound and presence of a heartbeat.

### Exclusion criteria

The following are the study exclusion criteria:

- Women already prescribed daily aspirin for more than 7 days;
- Women with multiple gestations;
- Fetal anomaly by ultrasound (Note: most fetal anomalies are not detectable by ultrasounds done at this early gestation. Subsequent discovery of a fetal anomaly is not an exclusion.);
- Hemoglobin < 7.0 g/dl at screening;
- Systolic blood pressure ≥ 140 and diastolic ≥ 90 at screening;
- Any other medical conditions that may be considered a contraindication per the judgment of the site investigator (e.g., Lupus, Type 1 Diabetes, hypertension, or any other known significant disease).

### Study procedures

The schedule of study procedures is outlined in Table 1.

**Table 1:** Timing of procedures

	6 0/7- 13 6/7 weeks GA	Bi-weekly after enrollment	4 weeks after enrollment	16-20 weeks GA	26-30 weeks GA	28-30 weeks GA	34 weeks GA	36 weeks GA until delivery	6 weeks Post-Partum	As needed
Screening										
Initial eligibility	●									
Ultrasound	●									
Consent	●									
Randomization/Enrollment	●									
Clinical Assessments										
Physical exam	●									
Medical history	●									
Use of other medicines	●	●								
Maternal Hb monitoring	●		●		●					
BP Monitoring	●			●		●	●	●		
Medication Activities										
Instructions for medication use	●	●								
Medication compliance^a^		●								
Resupply		●								
Outcome Assessment										
Preterm Birth									●	
SGA									●	
Preeclampsia/Eclampsia		●		●		●	●	●	●	●
Other maternal outcomes		●							●	●
Other fetal outcomes									●	●
Unanticipated medical care		●								●
Serious Adverse Events		●							●	●

^a^Medication is supplied until 36 weeks GA; therefore duration of medication-related monitoring depends on GA at randomization

#### Initial screening

Potential study participants will be recruited using multiple strategies so as to reach a diverse study population that includes nulliparous women who are pregnant. To reach the maximum number of potential participants, clinic-based and community-based recruitment methods will be used. Sites will determine the most effective method for their hospital/community.

Upon identification, a brief assessment of eligibility will be made to determine whether the patient is nulliparous and pregnant in the required GA window based on last menstrual period (LMP), has no more than two previous 1^st^ trimester pregnancy losses, and has no medical contraindications to aspirin or pregnancy. Knowledge of exact LMP and/or GA are not absolute contraindications to study enrollment, as ultrasound will ultimately determine the participant’s GA.

The initial screening will include collection of information on specific medical conditions, prior surgical procedures, medication use, allergy history, and outcomes of any prior pregnancies. If a contraindication to participation in the trial is found, the woman will be excluded from the trial at this point. If the field staff are uncertain about whether a woman is an appropriate candidate, her enrollment will be deferred until study investigators can review her medical history. Women who meet medical history eligibility criteria will proceed to have an ultrasound performed.

#### Ultrasound screening

To be included in the study, the following three findings must be obtained via ultrasound:

- A single intrauterine gestation
- Presence of a fetal heart rate
- Crown rump length (CRL) < 14 weeks gestation

Ultrasound will be assessed in all participants consistent with local good clinical practice. To obtain the CRL, a total of 3 measurements should be obtained in millimeters and the average should be utilized. Other measurements, such as the size of the gestational sac, will not be used. All study sonographers will be trained to assess gestational age using CRL measurements and quality will be monitored.

Since accurate information about LMP is frequently be unavailable in our prospective study population, gestational age will be assigned solely with the use of ultrasound measurement of CRL. The assignment of the projected due date will be consistent with the recent direction provided by the NICHD consensus statement [34] shown in Table 2.

**Table 2:** Guidelines for assigning projected due date

Gestational age by LMP	Difference between LMP and US GA	Basis for assigning projected due date
Unknown/Not available	N/A	CRL
<9 0/7 weeks	>5 days	CRL
	≤5 days	LMP
≥9 0/7 and ≤13 6/7 weeks	>7 days	CRL
	≤7 days	LMP

#### Clinical assessment

Each participant will be assessed to determine clinical status. The assessment will consist of a brief physical examination, medical history, and questions about the use of other medicines or medicinal products. It will also include a baseline hemoglobin (Hb) measurement. If the Hb measurement is < 7.0 g/dl, she will be excluded from the study.

#### Randomization procedures

Randomization of subjects will be carried out to obtain a 1:1 allocation ratio between the treatment and placebo arms. Randomization will be stratified by site. A computer algorithm generated by the data coordinating center (DCC) will create the random assignment to one of the treatment arms based on randomly permuted block design with randomly varied block sizes, known only by the DCC personnel.

### Study intervention and comparison

The study intervention is 81 mg of aspirin administered daily beginning between 6 0/7 weeks and 13 6/7 weeks through 36 0/7 weeks or delivery. Though 81 mg and 75 mg doses are both used as standard “low” doses in developing countries; the 81 mg dose was selected because it is consistent with the dosage used in other large trials such as EAGER, is the standard in five of the seven participating sites, and introduces no additional risk over the 75 mg dose. In fact, Duley et als meta-analysis showed a greater risk reduction for the development of preeclampsia associated with proteinuria in women treated with doses greater than 75 mg/day (RR 0.49, 95% CI 0.38-0.65) compared to lower doses (RR 0.86, 95% CI 0.79-0.93).^23^

Following randomization, each woman will be provided with a supply of either LDA or an identical appearing placebo. The drug or placebo will be enteric coated pills, provided in identical, child-resistant packaging, with written instructions for use. To ensure that literacy levels will not affect proper use, the study staff will provide verbal instructions at randomization and reinforce these guidelines at subsequent follow-up visits. Each woman will also receive a back-up supply of medication or placebo, which will be maintained throughout the duration of the study. The purpose of the backup is to bridge circumstances wherein the woman misses or is late to a planned follow-up visit or the primary supply of study drug/placebo is misplaced or destroyed. Medication compliance will be monitored through pill counts and the drug/placebo supply will be replenished during routine study visits.

### Blinding/masking

Both the aspirin and placebo will be procured from the same manufacturer. Packaging will be standardized across sites and will be labeled as ASA 81 mg/placebo, with the expiration data and a unique identifier.

Throughout the study, research staff and local health providers will be blinded to treatment status unless there is a serious adverse event potentially related to the treatment modality that requires un-blinding for safety reasons. One pharmacist at each site will remain unmasked to monitor randomization, drug supply, and safety as needed.

#### Monitoring

Participants will meet with study staff biweekly to monitor medical side effects and other medical co-interventions. As this is a pragmatic trial, no limitations on local treatment will be prescribed but rather simply documented. An assessment of unplanned medical visits will also be made at this time. Routine study visits will also provide an opportunity to monitor drug compliance and exchange her completed drug/placebo supply for a new allotment of medication. This process will be completed until the beginning of 36 0/7 weeks GA or the participant delivers.

In order to assess the development of preeclampsia, blood pressure will be monitored at the following time points:

- Between 16 and 20 weeks
- At 28–30 weeks
- At 34 weeks
- Every 2 weeks beyond that until delivery, alternating with pill monitoring visits

Each visit will consist of routine blood pressure measurements using a standardized blood pressure instrument and protocol. If the blood pressure is found to be >140/90, then proteinuria will be evaluated by urine dipstick, and the participant will be referred, as indicated.

### Primary outcome

The primary outcome of this study is preterm birth, which will be defined as delivery at or after 20 0/7 weeks and prior to 37 0/7 weeks. This will be determined based on actual date of delivery in comparison to the projected EDD, independent of whether or not the preterm delivery is indicated or spontaneous.

### Secondary outcomes

- Preeclampsia and eclampsia
- SGA newborn
- Perinatal mortality

### Other outcomes of interest

Maternal outcomes:

- Vaginal bleeding
- Antepartum hemorrhage
- Postpartum hemorrhage
- Maternal mortality
- Late pregnancy termination
- Change in maternal hemoglobin

Fetal and Neonatal outcomes:

- Preterm birth <34 0/7 weeks of pregnancy
- Birth weight <2500 g and <1500 g
- Fetal loss
- Spontaneous abortion
- Stillbirth
- Medical termination of pregnancy

### Safety monitoring

Aspirin remains the oldest prescribed drug in human medicine. As such, the safety profile has been well described. Large longitudinal studies of both adult populations and pregnant populations have been performed, with minimal or no side effects detected with the use of low doses. A 2014 systematic review of the use of LDA for the prevention of morbidity and mortality from preeclampsia found a 10% reduction in risk of preeclampsia, 20% reduction of SGA and a 14% reduction of preterm birth [35]. Further, there was no evidence of serious harms associated with LDA use during pregnancy. Bleeding-related complications, such as postpartum hemorrhage, maternal blood loss, and neonatal intracranial or intraventricular bleeding were not increased. The evidence on longer-term outcomes for offspring from in utero aspirin exposure (low-dose) is limited, but follow-up data from one large RCT is reassuring [35]. Nonetheless, it should be noted these studies have not assessed the safety of aspirin in pregnancy in the developing world where unique circumstances such as endemic anemia may be present. Therefore safety monitoring remains a strong focus of this project and can be divided into three distinct areas:

1. Active surveillance of maternal side effects and medical complications associated with aspirin: Maternal surveillance will be composed of active assessment of unintended medical visits. Likewise, where obtained for clinical care, hemoglobin will be recorded as well as administration of both oral and intravenous iron. The incidence of postpartum hemorrhage, antepartum hemorrhage, cesarean delivery and maternal death due to postpartum hemorrhage will also be monitored on an ongoing basis.
2. Evaluation of fetal side effects: If occurrences of major fetal abnormalities or fetal loss are discovered during ultrasound procedures or follow-up visits, they will be noted and the woman will be referred according to local standard of care. Likewise, stillbirth and late pregnancy loss (delivery between 16 and 20 weeks) will be monitored via established maternal and newborn health registry that is conducted within the Global Network infrastructure at each site. Fetal anomalies and loss will also be reviewed at least twice a year by the Data Monitoring Committee (DMC)
3. Evaluation of changes in maternal hemoglobin: Anemia will be monitored in all women, with sequential hemoglobin measurements at randomization and again between 26-30 weeks GA. Women with hemoglobin < 7.0 g/dl at randomization will be excluded from the study. Hemoglobin will also be measured at 4 weeks post-randomization in the first 500 available women who agree to participate, with changes in hemoglobin assessed by treatment group. Recognizing that site-specific characteristics may affect maternal hemoglobin, the 500 woman sample will include at least 50 women from each site. Women with hemoglobin levels of < 7.0 g/dL or with a change of more than 3.5 mg when measured post-randomization will be referred to a health provider to receive the local standard of care.

### Compliance monitoring

The participant will take daily LDA from the time of randomization until 36 0/7 weeks GA or delivery. Drug compliance will be routinely monitored throughout this period through pill counts and the completion of a medicine compliance form each time the pill supply is replenished. An interval medical obstetrical history will also be taken during these scheduled visits. Study staff will also provide reminders about proper drug administration and the importance of compliance during bi-weekly visits to monitor side effects.

### Site preparation

In preparation for study implementation, the sites will meet with health authorities and conduct community sensitization activities to ensure that study procedures are appropriate for the local context and to encourage commitment and engagement at the facility and community level. Site preparation activities will focus on:

- Identifying and hiring study staff;
- Developing site-specific procedures for safety monitoring (blood pressure, anemia) and procuring the necessary equipment;
- Exploring locally-acceptable methods to monitor and improve medication compliance;
- Identifying potential implementation challenges and developing culturally-appropriate solutions;
- Identifying local medicines/treatments that contain aspirin or have contraindications for its use;
- Educating health workers and community members on the use of aspirin in pregnancy (including safety of aspirin use).

### Potential risks and benefits to participants

There are several potential direct and indirect benefits of this trial. In LMIC, including those of the Global Network, the rate of preterm births can be as high as 19% of all deliveries amounting to an estimated 15 million preterm births in 2012 [2, 9]. Should this trial be successful and the rate of preterm deliveries be dropped by 20%, we could eliminate 3,750,000 of these early births per year. Aspirin has also been shown to decrease the incidence of stillbirth and delivery of SGA infants [21, 23, 24, 36]. In addition to a reduction of preterm birth, there is clear evidence of maternal benefit. There are data supporting the use of daily LDA to prevent preeclampsia [24, 37, 38]. This is an important benefit, as pregnancy induced hypertension (PIH) is a major contributor to maternal mortality [39].

Globally, preterm delivery remains a major health care financial burden [2, 40].

By reducing the number of preterm deliveries, the trial also has the potential to decrease need for extended and recurrent hospital stays that are frequent in this population, as well as impacting the cost of care for the long-lasting neurodevelopmental disorders and chronic health conditions incurred later on in life [5–7]. Moreover, with this simple strategy, we could potentially save the lives of over one million neonates that die due to prematurity every year [2].

The safety profile of aspirin is well-established and thoroughly investigated. Women with a known adverse reaction to acetylsalicylic acid (aspirin) will be excluded from the trial. This occurs rarely, but an allergic reaction to the intervention medication remains a small risk. Fetal concerns are also limited. Most notably, LDA has actually been shown to improve neurologic outcomes in preterm infants [29]. In light of such findings, the medical community acknowledges that antepartum exposure to low dose aspirin incurs only marginal fetal risk. Overall, the profound benefits of this intervention greatly outweigh the minimal risks to both mother and child.

### Analytical plan

#### Statistical analysis plan

Baseline demographic characteristics and key clinical measures will be compared between women in the two treatment arms using contingency table approaches for categorical variables and analysis of variance models and t-tests for continuous variables. For all of these analyses, comparisons will be made within each Global Network site and overall across the sites controlling for site in the models.

#### Primary analysis—risk of preterm (<37 weeks) birth

The primary analysis will compare the risk of preterm birth between the two treatment arms using two complementary approaches. First, the formal test of the primary hypothesis that the risk of preterm birth differs between the two arms will utilize a modified intention-to-treat approach based on a Cochran-Mantel-Haenszel (CMH) test stratified by Global Network sites. The approach is characterized as a modified intention to treat approach because the analysis population for the test will be all randomized pregnancies for which the delivery occurs after 20 weeks. Earlier deliveries (miscarriages) will be considered missing completely at random for purposes of this analysis.

In order to evaluate the sensitivity of the primary hypothesis test to the assumption by which early deliveries or losses are treated as randomly missing and to control for potential confounders of the treatment effect, additional analyses will be conducted using a series of three generalized linear models. These generalized linear models will be fit with the binary outcome of preterm (<37 week delivery) as the outcome measure. The initial model will include terms for treatment, site, and a treatment by site interaction. If the treatment by site interaction term is found to be significant (p < 0.05), then all subsequent models will include the interaction term and all effects will be reported by site. If the interaction term is not significant, it will be removed from the model and this initial model be used to estimate an unadjusted treatment effect controlling for site. The second series of models will include any demographic or clinical variable found to differ significantly between the treatment arms in the preliminary analyses described above. These models will be used to generate adjusted estimates of the treatment effect controlling for potential confounders. The third set of models will utilize extensions of the generalized linear model that incorporate inverse probability weighting to evaluate the sensitivity of the inference to treatment of miscarriages as randomly missing [41].

#### Secondary and exploratory analysis

This study has been designed to evaluate formally the differences between the two treatment arms for three secondary outcomes: the risk of perinatal mortality; the risk of eclampsia/preeclampsia, and the risk of a SGA infant. As with the primary analyses, each of these outcomes will be examined individually using formal tests of hypotheses that the risk differs between the two arms based on CMH tests stratified by Global Network sites. Analyses for each of these 3 secondary outcomes will utilize the same modified intention-to-treat population used for the primary outcome. Each of these hypothesis tests will be conducted at the 0.05 level of significance with no adjustment for multiple comparisons. In addition to this set of formal hypothesis tests, model based analyses comparable to those conducted for the primary analyses will be used to evaluate potential heterogeneity of treatment effect across sites and evaluate potential confounding. For each of the outcomes, an initial model will include terms for treatment, site, and a treatment by site interaction. If the treatment by site interaction term is found to be significant (p < 0.05), then all subsequent models will include the interaction term and all effects will be reported by site. If the interaction term is not significant, it will be removed from the model and this initial model be used to estimate an unadjusted treatment effect controlling for site. The second series of models will include any demographic or clinical variable found to differ significantly between the treatment arms in the preliminary analyses described above. These models will be used to generate adjusted estimates of the treatment effect controlling for potential confounders.

In addition to these planned formal secondary outcome analyses, we will also conduct exploratory outcomes for a number of other binary outcomes. The analytic approach for these exploratory analyses, will be comparable to the approach described above for the primary and formal secondary analyses. However, these exploratory analyses will focus on estimation of effect sizes and generation of hypotheses rather than on formal hypothesis tests. Initially, contingency tables will be used to generate estimates of the risk of each outcome for the two treatment arms, both overall and separately by Global Network (GN) site; these contingency tables will also be used to generate estimates of the relative risk associated with treatment by site and aggregated across sites adjusting for site. For each outcome a series of generalized linear models analogous to those described for the primary outcome will be fit to generate unadjusted estimates of risk, estimates of risk adjusted for potential confounders, and estimates of risk adjusted to account for any possible differences in risk between the arms associated with miscarriages.

### Sample size and power estimates

The risk of preterm birth < 37 weeks gestation in untreated nulliparous women was estimated to be about 8.0% for the EAGeR trial [15, 42]. Unpublished data for 2012 from the Global Network sites show preterm birth rates among sites, ranging from 2.9% to 9.8%, while WHO estimates the range for the countries in which Global Network sites are located to be 7.7% to 16.7% [2]. In developing sample size estimates we considered risks in the range of 8% to 14% and ultimately selected sample sizes based on a conservative estimate of 8%. Because the association of LDA and preterm birth may differ in international settings, we examined sample size requirements for reductions of 40%, 25%, and 20%. As shown in Table 3, the number of evaluable participants needed to detect a 20% reduction from a usual rate of 8% preterm births with 90% power is 5,483 per treatment arm. A conservative estimate of effect size was selected that would still be clinically relevant. To account for the loss of evaluable subjects due to spontaneous abortion, which are anticipated to occur in approximately 5% of participants) and loss to follow-up (assumed to be in the 1% to 2% range), sample sizes were increased by 8% to obtain a final sample size of 11,920 (or 5,960 per treatment arm).

**Table 3:** Evaluable sample size estimates

PTB rate in Placebo Arm	PTB rate in ASA	% Red.	Sample size per group	PTB rate in ASA	% Red.	Sample size per group	PTB rate in ASA	% Red.	Sample size per group
Power = 80% (α = 0.05; β = 0.2)									
8%	4.8%	40%	918	6.0%	25%	2554	6.40%	20%	4096
10%	6.0%	40%	721	7.5%	25%	2005	8.00%	20%	3213
12%	7.2%	40%	591	9.0%	25%	1638	9.60%	20%	2625
14%	8.4%	40%	497	10.0%	25%	1377	11.20%	20%	2204
Power = 90% (α = 0.05; β = 0.1)									
8%	4.8%	40%	1228	6.0%	25%	3419	6.40%	20%	**5483**
10%	6.0%	40%	965	7.5%	25%	2683	8.00%	20%	4301
12%	7.2%	40%	790	9.0%	25%	2193	9.60%	20%	3513
14%	8.4%	40%	665	10.5%	25%	1842	11.20%	20%	2950

### Sample size—secondary outcomes

One of the key secondary objectives of this trial is to evaluate the effect of low dose aspirin on perinatal mortality. We evaluated the reduction in perinatal mortality that could be detected with 80% power and 90% power under the assumption that the sample size for the study would be based on that needed to achieve the primary aim of demonstrating a 20% reduction in low-birth weight infants. The analyses were based on the assumption that the perinatal mortality rate in the Global Network population is approximately 45 perinatal deaths per 1000 deliveries [40]. With this estimated underlying mortality rate and 5,483 evaluable subjects per treatment arm required to demonstrate the 20% reduction in low birth weight prevalence as outlined in the primary aim, the study will have 80% power to detect a 24% reduction in perinatal mortality and 90% power to detect a 27% reduction in perinatal mortality.

To determine the sample size actually needed to demonstrate the reduction in perinatal mortality expected if the intervention results in a 20% reduction in prevalence of low birth weight infants, we examined both the prevalence of low birth weight infants across the Global Network sites and the perinatal mortality risk in both the low birth weight and normal birth weight infants. Based on 2013 MNH Registry data, the prevalence of low birth weight among all Global Network deliveries was 16.3% and the perinatal mortality rates were 20.5 per 1000 deliveries among normal birth weight infants and 162.3 per 1000 deliveries among low birth weight infants; these values yield a weighted average perinatal mortality rate of 43.6 per 1000 deliveries. If the LDA intervention is effective in reducing the prevalence of low birth weight deliveries by 20% (i.e. reducing the prevalence from 16.3% to 13%) and the conditional risk of perinatal mortality is unchanged in the low birth weight and normal birth weight cohorts, we would expect the resultant overall perinatal mortality rate to be reduced to 38.9 per 1000 deliveries or a 10.8% reduction. Detection of a reduction of this magnitude with 80% power would require an evaluable sample size of greater than 28,000 evaluable deliveries per treatment arm, and the study would need over 37,000 evaluable deliveries per arm to achieve 90% power to detect a reduction of this level in perinatal mortality.

Given the sample sizes associated with the second approach, the best alternative appears to be to power the study to demonstrate the 20% reduction in prevalence of low birth weight deliveries with a commitment to generate point and interval estimates of the impact of the intervention on perinatal mortality. By examining the risk of perinatal mortality in both the normal and low birth weight infants in the two arms, the trial would provide some insight about impacts of the intervention on mortality other than that mediated through the effect on birth weight.

We also conducted power analyses to examine the effect sizes that could be detected with the planned sample sizes for two additional secondary outcomes, prevalence of SGA infants and incidence of eclampsia/preeclampsia in the target population. Preliminary information from the 2013 Global Network MNH Registry indicates that the risk of each of these events in the population of interest is approximately 5%. Under the assumption of 5,483 evaluable participants in each treatment arm and an assumed Type I error rate of 0.05 for per-comparison analyses (i.e. no control for multiple comparisons in the analyses of these secondary outcomes), the study will have 70% power to detect a 20% reduction in the risk of each of these outcome measures, 85% power to detect a 24% reduction and 90% power to detect a 26% reduction. Consequently, the study is reasonably powered to examine these secondary outcome measures.

### Available population

The sites of the Global Network have access to approximately 68,000 deliveries annually with 24,000 being potentially eligible pregnancies for this study. Previous studies of interventions during pregnancy have experienced high rates of enrollment in this population. The Global Network sites estimate that between one third and one half of eligible women will enroll in this protocol, which will allow the study to meet recruitment goals within 18-24 months.

### Projected recruitment time

Each site will begin recruitment after their site-specific regulatory approvals are in place, the site has been adequately prepared, and training is completed. This will require a staggered start, as the timeline for these activities will vary per site. The projected timeline for this study is approximately 3 years. This includes a 12 month preparatory phase with 3 months for training, an 18 month recruitment period, and 6 months for data cleaning and analysis (Table 4).

**Table 4:** Study timeline

	YR 1				YR 2				YR 3				YR 4	
	Q1	Q2	Q3	Q4	Q1	Q2	Q3	Q4	Q1	Q2	Q3	Q4	Q1	Q2
Preparatory Activities														
Document Development (Protocol, forms, etc.)	●	●	●	●										
Procurement of medication and other materials	●	●	●	●										
Approvals (IRB, ERC, Drug Authorities, etc.)	●	●	●	●										
Site Preparation			●	●	●	●								
Training				●	●	●								
Recruitment (24 months)					●	●	●	●	●	●	●	●		
Data Cleaning and Analysis (6 months)													●	●

### Study monitoring plan

#### Serious adverse events

Serious Adverse events (SAEs) will be monitored continuously using a special form that will be required for any event that meets the following criteria:

- Results in death;
- Is life-threatening;
- Requires hospitalization or prolongs existing hospitalization;
- Results in persistent or significant disability or incapacity;
- Any other serious or unexpected adverse event that the study investigator(s) feels should be reported.

The specific adverse events to be monitored in this trial include maternal death, GI bleeding (vomiting blood), fetal anomaly, gastroschisis, post-partum hemorrhage, or antepartum hemorrhage.

#### Data monitoring plan and stopping rules

All Global Network sites will report data to the Global Network Data Coordinating Center (DCC) (RTI International). Data will be used to evaluate protocol adherence and site performance (e.g., recruitment, loss to follow-up, data quality). The DCC will provide standardized progress reports to NICHD and site investigators on a monthly basis to monitor outcome variables and adverse events.

Oversight of the trial will be handled by two principal groups with different focuses:

1. Protocol-focused Steering Committee (SC): The SC is comprised of the Central Study Team from Christiana Care and Jawaharlal Nehru Medical College (JNMC), NICHD, the DCC, and investigators from each of the participating sites (see Table 5). The Central Study Team, with assistance from NICHD and the DCC, will have primary responsibility for overall study design, development of study materials and procedures, and oversight of study implementation. They will meet via conference call bi-weekly to monitor study progress and ensure proper implementation of the trial. The site Investigators will be responsible for providing guidance on study design, developing site-specific implementation plans, ensuring study staff are properly trained, and providing oversight of the study at the site level. The SC will convene via conference call at least once per quarter and will meet in person twice a year to discuss study design and implementation issues. Members of the Central Study team, NICHD, and RTI will also conduct site visits, as the budget allows, to bolster enthusiasm, provide hands-on training and education to the participating staff, and address site-specific issues, if any.

**Table 5:** ASPIRIN Study Team Members

Central Study Team: Global Network
Site 08 (Belgaum, India)
*Richard Derman, MD, MPH Principal Investigator, Thomas Jefferson University, Philadelphia, PA USA*
Bhalchandra Kodkany, MD, MBBS Senior Foreign Investigator, KLE University’s JN Medical College, Belagavi, India
Shivaprasad S. Goudar MD, MHPE Co-Investigator, KLE University’s JN Medical College, Belagavi, India
Matthew K. Hoffman, MD, MPH Lead Investigator for ASPIRIN Protocol, Christiana Care, Newark, DE USA
Mrityunjay C. Metgud, MD ASPIRIN Country Coordinator, KLE University’s JN Medical College, Belagavi, India
Frances Jaeger, MA, DrPhCo-Investigator, Thomas Jefferson University, Philadelphia, PA USA
Amit Revankar Data Manager, KLE University’s JN Medical College, Belagavi, India
Data Coordinating Center - RTI International Elizabeth McClure, PhD Principal Investigator Data Coordinating Center, RTI International, Research Triangle Park, NC USA
Dennis Wallace, PhD Senior Statistician, RTI International, Research Triangle Park, NC USA
Norman Goco, MHS Protocol Manager, RTI International, Research Triangle Park, NC USA
Jay Hemingway-Foday, MPH, MSW Protocol Manager for ASPIRIN Protocol, RTI International, Research Triangle Park, NC USA
Steve Litavecz, MBA Data Manager, RTI International, Research Triangle Park, NC USA
Janet Moore, MS Statistician, RTI International, Research Triangle Park, NC USA
Emily MacGuire, MPH Study Coordinator, RTI International, Research Triangle Park, NC USA
*Eunice Kennedy Shriver *National Institute of Child Health and Human Development (NICHD)
*Marion Koso-Thomas, MD MPH, Medical Officer, Global Network for Women's and Children's Health Research, NICHD, Washington, DC USA*
Menachem Miodovnik, MD Medical Officer, Global Network for Women’s and Children’s Health *Eunice Kennedy Shriver* National Institute of Child Health and Human Development (NICHD)
**Site Investigators**
Global Network Site 02 (Democratic Republic of Congo)
Carl L. Bose, M.D. Principal Investigator, University of North Carolina School of Medicine
Antoinette Tshefu, M.D, Ph.D., M.P.H Senior Foreign Investigator, Kinshasa School of Public Health, Kinshasa, Democratic Republic of Congo
Adrien Lokangaka, M.D., M.P.H. Country Coordinator, Kinshasa School of Public Health, Kinshasa, Democratic Republic of Congo
Daniel Ishoso, MD ASPIRIN Coordinator, Kinshasa School of Public Health, Kinshasa, Democratic Republic of Congo
Global Network Site 06 (Guatemala)
Nancy Krebs, MD Principal Investigator, University of Colorado Health Care System (UCHCS)
K. Michael Hambidge, MD Co-Investigator, University of Colorado Health Care System (UCHCS)
Ana Garces, MD, MPH Senior Foreign Investigator, Instituto de Nutrición de Centroamérica y Panamá (INCAP), Guatemala City, Guatemala
Lester Figueroa, MD Country Coordinator, Instituto de Nutrición de Centroamérica y Panamá (INCAP), Guatemala City, Guatemala
Maynor Manrique, Data Manager, INCAP, Instituto de Nutrición de Centroamérica y Panamá (INCAP), Guatemala City, Guatemala
Global Network Site 09 (Pakistan)
Robert L. Goldenberg, MD Principal Investigator, Columbia University
Sarah Saleem, MD Senior Foreign Investigator, Aga Khan University, Karachi, Pakistan
Saleem Jessani ASPIRIN Coordinator, Aga Khan University Karachi, Pakistan
Zaheer Habib Data Manager, Aga Khan University, Karachi, Pakistan
Global Network Site 11 (Nagpur, India)
Patricia L. Hibberd, MD, PhD Boston University School of Public Health
Archana Patel, MD, DNB, MSCE Senior Foreign Investigator, Lata Medical Research Foundation (LMRF), Nagpur, India
Prabir B. Das, MD ASPIRIN Country Coordinator, Lata Medical Research Foundation (LMRF), Nagpur, India
Amber Prakash, Data Manager, Lata Medical Research Foundation (LMRF), Nagpur, India
Global Network Site 03 (Zambia)
Wally Carlo, MDPrincipal Investigator, University of Alabama at Birmingham
Elwyn Chomba, MBChB, DCH, MRCPSenior Foreign Investigator, University Teaching Hospital, Lusaka, Zambia
Musaku Mwenechanya, MD Country Coordinator, University Teaching Hospital, Lusaka, Zambia
Cindy Chirwa ASPIRIN Coordinator, University Teaching Hospital, Lusaka, Zambia
Ernest Banda Data Manager, University Teaching Hospital, Lusaka, Zambia
Global Network Site 12 (Kenya)
Edward A. Liechty, MDPrincipal Investigator, Indiana University School of Medicine
Fabian Esamai, MBChB, MMed, PhDSenior Foreign Investigator, Moi University School of Medicine, Eldoret, Kenya
Emmah Achieng ASPIRIN Coordinator, Moi University, Eldoret, Kenya
Kevin Otieno Data Manager, Moi University, Eldoret, Kenya
Technical Advisory Group
Robert M. Silver, MD University of Utah Health Sciences Center
Enrique Schisterman, Ph.D Chief and Senior Investigator Epidemiology Branch, DIPHR *Eunice Kennedy Shriver* National Institute of Child Health and Human Development
Rob Nathan, MD Ultrasound Quality Control Advisory, Department of Radiology, University of Washington, Seattle, WA
2. Data Monitoring Committee (DMC): The DMC, a standing group that monitors all NICHD-funded Global Network studies, will be responsible for ensuring safe and ethical treatment of study participants through monitoring of the study. The membership will include, at a minimum, a statistician, obstetrician, pediatrician and an expert in international health. The DMC designated by NICHD will review the data collected at approximate 6 month intervals throughout the course of the study. The DMC reports, which are prepared by the Data coordinating center, will include information on study enrollment rates and participant progress through the study, participant compliance with protocol-specified treatment regimens, protocol violations, adverse events, and efficacy outcomes. The focus of the DMC review will be on monitoring participant safety and study progress/futility but data on treatment effectiveness will also be presented to frame the DMC discussions on safety and futility. However, no formal interim analysis of efficacy or effectiveness are planned and the DMC will not be responsible for stopping the trial for efficacy. The DMC will be charged with monitoring adverse events and side effects from LDA. All known associated side effects and specific obstetric or fetal concerns will be considered reportable to the DMC. The study will be reviewed by the DMC bi-annually at a minimum, but may be reviewed more frequently if concerns are raised about participant safety or about adequate process of the study.

### Data management procedures

Data will be collected both prospectively and from existing clinical records, using hard copy forms or Android Tablets. Regardless of data capture methodology, all data will be kept confidential. Each participant will be assigned a unique study ID which will be used to identify the participant. Only the screening log will contain the name (which is not transmitted). When hard copy forms are used, they will be retained in a secure location for possible editing or queries at the central data entry site. Data will be entered into computers using the Data Management System (DMS) developed by RTI and the assigned study number. The DMS will also allow site staff to produce project reports and backup the study database. Electronic data will be transferred from each data management computer to a single Research Unit Data Center (RUDC) in each country, creating a complete data repository. At least once per week, data will be transmitted from the RUDC to the DCC. The data center will conduct training on the DMS system, as needed, and will maintain the central database for the study.

Precision and accuracy of actual data collected will be assessed by chart review (random 5%) and internal procedures using the computer program. Monthly audits and incomplete data reports will be performed by a review team consisting of at least of the SFI and the country coordinator. Data editing and error resolution will be performed monthly. In addition, a sample of participants will be visited to confirm their participation, with procedures determined per site. These activities will be shared between the site and the data center.

### Quality control

#### Training

All study personnel must participate in training on the proper implementation of study procedures and the ethics of conducting research with human subjects before beginning any research activity. The SFI and project coordinator will ensure that all study personnel receive appropriate training, and obtain required certification ensuring that they have met training objectives. RTI will be responsible for developing a certification test. The SFI and project manager will be responsible for overseeing the certification process.

#### Study monitoring

Major monitoring responsibilities of the PI/SFI, assisted by the country coordinator, are (1) confirming proper IRB approval; (2) monitoring the delivery of the study intervention; (3) assessing and evaluating the quality of study implementation; (4) ensuring compliance with the intervention, including proper randomization; (5) evaluating accuracy, precision, and completeness of data collected, entered, and transmitted (along with the DCC); (6) ensuring that all personnel are fulfilling their obligations; (7) maintaining morale and enthusiasm of the staff; (8) handling ad hoc problems and maintaining communication; (9) ensuring inter-site consistency; and (10) proposing improvements to the monitoring activities.

NICHD and the DCC staff will conduct site visits as needed. These visits will include review of individual participant records, including supporting data, to ensure protection of study participants, compliance with the protocol, and accuracy and completeness of records. The SFI/PI will make study documents (e.g., logbooks, data forms, staff training certificates) and pertinent hospital/clinic records readily available for inspection by the local IRB, site monitors, and the NICHD for confirmation of the study data.

#### Drug quality assurance and monitoring

The study drug manufacturer will have a Good Manufacturing Practices (GMP) designation vetted by the FDA and a certificate of authenticity will be provided. Each site will adapt best practice guidelines for drug shipment and storage to the needs and infrastructure of their local environment. Study staff will be trained in on the drug shipment and storage plan to ensure that best practices are maintained at all time. Additionally, participants will receive detailed instruction on proper storage of the study drug at home. Drug stability information will be maintained throughout the study. For quality assurance, a sample of pills from each site will be randomly selected and tested for bioavailability at multiple time points during the study period. A sample from each batch will be tested.

#### Ultrasound gestational age dating

All study sonographers will be certified to assess gestational age using CRL measurements. A sample of ultrasound images at each site will be reviewed by study investigators to ensure that CRL is accurately measured and gestational age is accurately assigned. If discrepancies are found, the sonographers will be retrained. Ultrasound will be used only for pregnancy dating and no other purpose.

## Discussion

This study will assess the impact of initiation of LDA between 6 and 14 weeks of gestation. Though several large studies have examined the effects of aspirin on pregnancy, this study is unique because it will examine this treatment early in pregnancy in low-middle income countries with preterm birth as a primary outcome. Though studies of LDA initiated later in pregnancy [31] have consistently shown benefit, the extant of impact may be muted by the fact that late initiation may fail to protect the placenta as it undergoes cellular division and invests itself into the maternal decidua. Secondary analyses have consistently suggested that the effect size is markedly increased when LDA is initiated prior to 16 weeks; however, this will be the first study to examine this question on this scale. The importance of developing low-cost, high impact interventions in low-middle income countries is magnified as they are often unable to bear the financial costs of treating illness. Likewise this approach examines the important question of whether the biologic underpinning diseases are similar between the developed and developing world.

## Abbreviations

ACOG: American congress of obstetricians and gynecologists
APS: Antiphospholipid syndrome
ASA: Acetylsalicylic acid
BP: Blood pressure
CLASP: Collaborative low-dose aspirin study in pregnancy
COX: Cyclo-oxygenase
CRL: Crown rump length
DCC: Data coordinating center
DMC: Data monitoring committee
DMS: Data management system
DRC: Democratic Republic of Congo
EAGER: Effects of aspirin in gestation and reproduction study
EDD: Estimated due date (or estimated date of delivery)
ERC: Ethical review Committee
GA: Gestational age
GN: Global network for women’s and children’s health research
Hb: Hemoglobin
IRB: Institutional Review Board
IUGR: Intrauterine growth restriction
JNMC: Jawaharlal nehru medical college
LBW: Low birth weight
LDA: Low dose aspirin
LMP: Last menstrual period
MNH: Maternal and newborn health
NICHD: Eunice Kennedy Shriver National institute of child health and human development
NIH: National Institutes of Health
NSAID: Non-steroidal anti-inflammatory drug
PIH: Pregnancy induced hypertension
PMNCH: The Partnership for Maternal, Newborn, & Child Health
PROM: Premature rupture of membranes
PTB: Preterm birth
RCT: Randomized controlled trial
RTI: Research triangle institute international
SAE: Serious adverse event
SFI: senior foreign investigator
SGA: Small for gestational age
SPTB: Spontaneous preterm birth
VLBW: Very low birth weight
WHO: World Health Organization
